# Editorial: On the cusp of the silent wave of the long COVID pandemic: why, what and how should we tackle this emerging syndrome in the clinic and population?

**DOI:** 10.3389/fpubh.2024.1483693

**Published:** 2024-10-23

**Authors:** Francisco Westermeier, Nuno Sepúlveda

**Affiliations:** ^1^Department of Health Studies, Institute of Biomedical Science, FH Joanneum University of Applied Sciences, Graz, Austria; ^2^Centro Integrativo de Biología y Química Aplicada (CIBQA), Universidad Bernardo O'Higgins, Santiago, Chile; ^3^Faculty of Mathematics and Information Science, Warsaw University of Technology, Warsaw, Poland; ^4^CEAUL-Centro de Estatística e Aplicações da Universidade de Lisboa, Lisbon, Portugal

**Keywords:** post-COVID-19 syndrome, long COVID, post-acute sequelae of SARS-CoV-2, post-acute coronavirus (COVID-19) syndrome, quality of life, healthcare, Myalgic Encephalomyelitis/Chronic Fatigue Syndrome

## 1 Context

There is an urgent public health problem due to the rising number of individuals who remain with their health and daily functions impaired for months and even years after a SARS-CoV-2 infection (1). This impairment is encapsulated by a new medical condition known as post COVID-19 syndrome, post-acute COVID-19 syndrome, post-acute sequelae of SARS-CoV-2 infection, and persistent post-COVID-19 syndrome. The general public knows this condition as long COVID (LC), a coined termed by patients at the beginning of the pandemic (1).

Individuals with LC report experiencing many symptoms, including fatigue, post-exertional malaise (PEM), and sleep disturbances (2). Coincidently, these specific symptoms are the heart of the most consensual case definitions of Myalgic Encephalomyelitis/Chronic Fatigue Syndrome (ME/CFS), a “older” disease often triggered by an infection (e.g., infectious mononucleosis) and also causing high levels of physical and mental distress (3). It is then no surprise that individuals with LC can also receive an ME/CFS diagnosis (4, 5). This diagnostic overlap is the main reason for the growing interest in understanding the medical relationship between LC and ME/CFS in order to accelerate the development of efficacious pharmacological and non-pharmacological interventions for the benefit of the patients (6–8).

The present Research Topic aimed then at gathering new data on the public health and medicine of LC and ME/CFS. The Research Topic compiled 11 papers of which nine were original research. Seven papers concerned LC directly or indirectly. The remaining four papers focused on ME/CFS specifically or together with LC. Below the reader can find a brief account of the research conducted.

## 2 Contributions to current knowledge on the public health impact of LC

Four large-scale studies on LC surveyed more than 1,000 individuals. These studies evaluated different health-related metrics after a SARS-CoV-2 infection using retrospective data or convenient cross-sectional surveys.

From the United States of America, Sandoval et al. reported a retrospective study on 91,007 adult patients from Southeast Texas. The study aimed at evaluating the chance of hospital readmission after a SARS-CoV-2 infection. The main finding is that 21% of the individuals were readmitted to the hospital within 90 days after infection. The chance of hospital admission seemed to be dependent on different factors, including a dose-response relationship with area deprivation index.

From 16 countries in Latin America, with the special focus on Ecuador, Mexico, Argentina, Colombia, Peru, and Chile, Angarita-Fonseca et al. estimated the prevalence of individuals with LC using an online survey of 2,466 people. In this survey, 1,178 individuals (47.8%) reported experiencing symptoms after 3 months of a SARS-CoV-2 infection. This survey also suggested several risk factors for LC, including a COVID-19 episode earlier in the pandemic, old age, no vaccination against SARS-CoV-2, and a high number of pre-existing co-morbidities.

From Brazil, Malheiro et al. conducted a telephone-based survey in the city of São Paulo on 291 hospitalized and 1,118 non-hospitalized patients with COVID-19. The study also aimed at estimating the prevalence of LC at least 3 months after infection and to determine the respective risk factors. The study estimated the LC prevalence at 47.1 and 49.5% for these two populations, respectively. These estimates were in almost perfect agreement with the ones reported by Angarita-Fonseca et al.. Again, pre-existing co-morbidities such as hypertension are possible risk factors for LC manifestations.

From Italy, Gagliotti et al. estimated the incidence and determined the factors affecting the access to specific healthcare services up to a year after the acute phase of a SARS-CoV-2 infection. The study was conducted in a large number of healthy individuals (*n* = 35,128 and 88,881 from Emilia-Romagna and Veneto, respectively). This study found that more than 20% of the surveyed individuals accessed a health service, mostly outpatient care more than drug prescription as follow-up of their SARS-CoV-2 infection. Whether this access was a direct cause of LC specifically remained an open question from this study.

The three remaining studies on LC contemplated a moderate number of surveyed individuals. From Castellón in Spain, Pérez Catalán et al. provided evidence that the quality of life of 486 Spanish patients tended to remain affected after 1 year of their COVID-19-related hospitalization. This particular study was already criticized due to its reliance on telephone interviews (9). From Vancouver in Canada, Magel et al. followed up 88 patients previously hospitalized due to COVID-19 complications. This study focused on how the levels of fatigue evolved over time. The study that 67% (*n* = 58) of individuals experienced fatigue at 3 months post-infection, but this percentage dropped to 60% (*n* = 47) after 6 months. The same drop was observed in patients experiencing substantial fatigue (16–6% after 3 and 6 months after infection, respectively). Accordingly to other studies published in this Research Topic, the study also provided evidence for a positive association between the number of pre-existing comorbidities and fatigue. From Bari in Italy, Resta et al. reported the single study conducted in a clinical setting. The study focused post-COVID exertion dyspnoea in 318 patients at 3 months after SARS-CoV-2 infection. In this study, the study participants performed a 6-min walking test after which 59.7% showed evidence of dyspnoea. This finding showed that exertion dyspnea might be part of the PEM spectrum in LC.

## 3 Research on ME/CFS with possible implications to LC

Four papers concentrated their attention on ME/CFS with possible implications to LC. For example, the new study of Hannestad et al. provided evidence for an increase of IgG antibodies against human adenovirus after a SARS-CoV-2 infection in a Swedish cohort of patients with ME/CFS. This finding suggested that a SARS-CoV-2 infection could prompt the reactivation of the human adenovirus. Such a reactivation might explain the worsening of symptoms in some patients with ME/CFS after a SARS-CoV-2 infection, as suggested elsewhere (10). Another example is the perspective paper of Scheibenbogen et al. who compiled and discussed a list of candidate drugs that could treat both ME/CFS and LC patients. This perspective paper also provided an important concept for developing clinical trial networks in this era of LC and ME/CFS. In turn, Grabowska et al. discussed the concept of extending current large-scale prevalence studies of LC to ME/CFS, a disease whose incidence and prevalence remain largely elusive (11). According to these authors, estimating ME/CFS prevalence comes at a minimal cost in such studies, but requires the recognition of PEM as one of the cardinal symptoms for ME/CFS diagnosis. The recognition of PEM in medical care is also important, as demonstrated by a new study of Wormgoor and Rodenburg on a cohort of Norwegian ME/CFS patients. However, data from this new study suggested that PEM remains a neglected symptom by specialized medical staff and healthcare providers.

## 4 Two final remarks

Most of the new contributions published in this Research Topic were based on the evaluation of simple metrics aiming at capturing different sequelae facets of a SARS-CoV-2 infection. These metrics are fundamental to understand the impact of the problem on public health and society, as reviewed elsewhere (1). At the same time, the abundance of descriptive studies suggested that we are still at the early stage of addressing the LC problem. In this scenario, the great benefit of this Research Topic seemed to come from an integrated collection of papers where LC and ME/CFS are somehow put side-by-side. This is the case of Scheibenbogen et al. who aimed at leveraging pre-existing knowledge on ME/CFS pathogenesis and treatment with a potential impact on the healthcare of LC patients.

All the original research articles published in this Research Topic had the curiosity of coming from studying European, North American, and South American populations. This illustrates the wide extension of the LC challenge across the world. However, no papers from Asia and Africa were published in this Research Topic. In the case of Asia, it was a simple coincidence with several papers being submitted, but subsequently rejected for one reason or another. This contrasted with Africa from which no submission was received. Interestingly, the current prevalence estimates of LC in Africa (12) are similar to the ones found by Malheiro et al. in São Paulo Brazil and Angarita-Fonseca et al. across Latin America. Given this statistical coincidence, we had the expectation to collect some research studies on LC from this continent. Does this mean that the interest on LC is fading away in Africa even if there is evidence for an accumulation of cases elsewhere? Perhaps this is the right time for revitalizing the LC research in Africa to determine whether this continent is an exception in the global burden of this new post-pandemic condition.
